# Provider and facility readiness for age-friendly health services for older adults in primary health care centres in southwest, Nigeria

**DOI:** 10.1371/journal.pgph.0001411

**Published:** 2023-08-08

**Authors:** Adedoyin O. Ogunyemi, Mobolanle R. Balogun, Adedayo E. Ojo, Sarah B. Welch, Oluwatosin O. Onasanya, Victoria O. Yesufu, Abisola O. Omotayo, Lisa R. Hirschhorn

**Affiliations:** 1 Department of Community Health and Primary Care, College of Medicine, University of Lagos, Lagos, Nigeria; 2 Department of Internal Medicine, University of Abuja Teaching Hospital, Abuja, Nigeria; 3 Department of Epidemiology and Global Health, University Medical Centre, Utrecht University, Utrecht, The Netherlands; 4 Buehler Center for Health Policy and Economics, Feinberg School of Medicine, Northwestern University, Chicago, IL, United States of America; 5 Robert J Havey Institute of Global Health, Feinberg School of Medicine, Northwestern University, Chicago IL, United States of America; 6 Lagos State Ministry of Health, Lagos, Nigeria; 7 Lagos State Sports Commission, Lagos, Nigeria; 8 Department of Medical Social Sciences, Feinberg School of Medicine, Northwestern University, Chicago IL, United States of America; African Population and Health Research Center, KENYA

## Abstract

There is a growing focus on interventions at the health system level to promote healthy aging and provide age-friendly health services (AFHS) in low- and middle-income countries where populations are aging. This study aimed to determine the provider and facility readiness for AFHS. We developed and implemented surveys to collect PHC facility capacity and readiness to deliver AFHS and a KAP survey for facility healthcare workers based on guidelines from the WHO age-friendly tool kit and questionnaires from other studies. Direct observation and structured interviews of facility heads were conducted in a stratified random sample of 15 out of the 57 comprehensive PHC facilities in Lagos, Nigeria. One hundred and twenty providers were conveniently sampled for the KAP survey. Statistical analysis was conducted using STATA version 15 (StataCorp, College Station, Texas, USA). For facility readiness, only 13.3% of PHCs sometimes offered hearing assessment and none of the PHCs offered colorectal cancer assessment. Few (20.0%) facilities offered home services and only 1 (0.7%) had dedicated funding for care of older people. Ramps were at the entrance in 60.0% of facilities and almost half (43.3%) of the PHCs had wheelchair accessible entrances to the public toilets. The majority of HCWs (81.7%) had heard about healthy aging but only 5.0% about AFHS, only 10.8% reported formal training. Around a third knew about specific conditions which affect people as they age, including; depression (37.5%), urinary incontinence (35.0%), and falls/immobility (33.3%). Over half of the providers (54.2%) screened for malnutrition in older patients, 25.8% screened for suspected elder abuse and much less (19.2%) for delirium. This study found some areas of strength but also gaps in facility readiness as well as knowledge and training needed to support AFHS care. We recommend identifying interventions to improve the availability and delivery of care for older adults.

## Introduction

Aging of the population is occurring globally but more rapidly in low- and middle-income countries (LMICs) countries such as Nigeria [1]. According to the Nigerian Population Commission, older adults, defined as those aged 60 years and above, are projected to rise from 9.4 million in 2020 to 16 million by 2030 and 47 million by 2060 representing an increase in proportion from 4.5% to 10% [2–4]. There is also a recognition of the growing burden of chronic non-communicable diseases (NCDs) associated in part with this aging and other age-related conditions. This highlights the need for interventions to promote healthy aging and reduce the incidence, morbidity and mortality associated with NCDs [5].

Primary health care is the community entry point for the healthcare delivery system for many Africans, particularly Nigerians, and even more so for those who live in underserved areas [6, 7]. It has been shown that investments in PHC improve equity and access, health care performance, accountability of health systems, and health outcomes [8]. However, weak implementation of primary health care in Nigeria has been well documented, leading to a PHC revitalization process by the Federal Ministry of Health (FMOH) in 2017 [7–9].

According to the 2018 Astana declaration, effective and people-centered PHC is critical to ensuring that everyone everywhere is able to enjoy the highest attainable standard of health and achieve the global goals of Universal Health Coverage (UHC) [6]. Until recently, there were no documented nor functional policies on the care and welfare of older adults in Nigeria. In 2021, the Federal Executive Council (FEC) approved the National Policy on Ageing for Older Persons in Nigeria [10]. A 10-year (2022–2032) strategic roadmap was developed based on this policy with two main goals (i) to mainstream aging into existing multi-sector programs, services, implementation plan and budget lines and (ii) initiating, piloting and replicating new opportunities [11]. However, it is in its early stages with no notable changes. In addition, PHC services are poorly organized, weak and not well designed to meet the complex health needs of older adults [12]. The challenges faced by older adults in accessing care can be further worsened by conditions which impact mobility and independence [13]. Despite agreeing to achieve the United Nations Sustainable Development Goals (SDGs) and the mantra of “leaving no one behind” by 2030, the inclusion older persons in the agenda implementation has not been considered a core development priority in Nigeria.

In response to the growing recognition of the need to improve care for aging adults, the World Health Organization (WHO) developed a guideline for providing Age-Friendly Health Services (AFHS) at PHCs in 2008 [14]. The guideline intends to improve PHC service delivery for older individuals through structural changes and educating PHC workers about the specific needs of these individuals while more broadly raising awareness of the health conditions experienced by this population. To achieve the goals, the document provides guidance on how to make PHC management procedures more responsive to the needs of older adults and how to undertake environmental audits to test age-friendliness [6, 14]. WHO defined three areas in which age-friendly principles need to be applied: information, education, communication, and training; health care management systems; and the physical environment [14].

A systematic review based on the WHO age-friendly principles found that the benefits of implementation in health care settings included a decrease in hospital readmission rates and a reduction in the four main geriatric syndromes: episodes of falls, delirium, immobility, and incontinence [15]. Despite these findings, the barriers to implementation include a lack of human, financial and economic resources in most settings [16–18]. Other barriers include knowledge and formal training as well as the need for structural changes in the physical environment of health facilities such as ramps, wheelchair-accessible bathrooms, and ensuring a safe environment [14]. In the United States, majority of the clinicians acknowledged the benefits of AFHS but reported limited knowledge of the specificities, however, screening for depression and review of high risk medication use were among the leading age-friendly services provided [19]. Findings from an Ugandan study on health systems readiness for age-friendly readiness revealed low readiness due to poor scores in leadership, financing, human resources and health management information system [20]. A study of older client’s perspectives towards an age-friendly hospital in the University of Benin Teaching Hospital, Nigeria, found most participants were satisfied with the services provided but highlighted few trained health personnel and infrastructural gaps [21].

Lagos State, a mega-city located in Southwest Nigeria, has been at the forefront of health sector development working to achieve Universal Health Coverage (UHC) by embarking on reforms to strengthen the PHCs in the State including the implementation of the new policy on PHC revitalization [22]. Current estimates of the older adult population in Lagos show that 4.5% or about 787,500 individuals would benefit from AFHS [4, 23]. To the best of our knowledge, there are no studies done so far to determine the age-friendliness of PHCs in Lagos State. This research fills that gap by determining the knowledge, attitudes and experience of PHC care providers in the delivery of AFHS and assessing facility readiness and the level of implementation of the WHO guideline for age-friendly PHCs.

## Materials and methods

### Setting

Nigeria is the most populous country in Africa with a population of over 200 million and is divided into six geopolitical zones. Nigeria’s PHC system is run through a 5-tier system consisting of medical officers of health who supervise the centres, nurse practitioners and community health officers who serve as the head of the PHCs and supervise community health extension workers (CHEWS) [24]. Lagos State is located in the southwestern zone of Nigeria with an estimated population of 17.5 million [23]. The state has 5 administrative divisions, 57 councils, 376 wards, and over 300 PHCs [25]. At least 57 of the PHCs in the state are comprehensive PHCs, which are staffed by medical doctors and open for 24 hours. The routine primary health services are under the responsibility of the local government [26].

### Study design and population

We conducted a cross-sectional study among a stratified sample of comprehensive PHC facilities and their health providers to understand facility readiness and provider knowledge, attitudes, experiences to provide age-friendly primary care in Lagos. A proportionate sampling was used to select the 15 PHCs used in the study, with sample size determined by resources available for the study. The stratified random sample of 15 was obtained from the 57 councils across the five administrative divisions of Lagos State using the formula (sample size/population size) x stratum size. In selecting the comprehensive PHCs to be used, simple random sampling was employed utilizing a table of random numbers.

To determine the knowledge, attitude and practice of the delivery of AFHS among the health providers, we received a list of care providers from the facility head at each PHC. We did a convenience sample of 8 HCW present during the visit. If more than 8 were present, we did sequential sample in, while if fewer were present, we visited as many times until 8 health workers were interviewed to achieve a total of 120 participants. Eligible providers included doctors, nurses, community health officers and CHEWs and laboratory staff who were present at the time of the facility survey and consented to complete the HCW survey. We had an acceptance rate greater than 95% in all cadre of healthcare workers in the PHCs visited.

### Survey design

An interviewer-administered Knowledge, Attitudes and Practices (KAP) survey was developed by the team using the guidelines from the WHO age-friendly tool kit and questionnaires from other studies that assessed healthcare worker readiness to provide age-friendly health services [14, 27, 28]. The survey captured knowledge, attitudes and practices in the delivery of AFHS as well as relevant demographics and professional experience. Questions were asked using a 4-point (attitude) or 5-point (practice) Likert scale. The main dependent variables were the knowledge, attitude and practice scores while the independent variables included participants demographic characteristics and work experience with older adults. A pilot test of the survey was done in four PHCs in Lagos State after we had selected the study sample and we adapted for clarity and relevance based on feedback.

The facility readiness survey was developed from the WHO’s toolkit and guidelines for age-friendly PHCs [14]. Readiness was measured in five areas: geriatric care training and service delivery; availability of clinical services and health assessment for older persons; facility design and physical environment; and facility health management information system.

### Data collection

A total of six trained research assistants collected data via interviews and were paired for the assessment of facilities and the involvement of a third observer. The facility data was collected through direct observation and a structured interview of the head of the PHC to complete the assessment.

### Data management and analysis

To ensure data quality, the data were entered into an excel sheet by two different data entry officers and compared for correctness before being exported for analysis. When there was a discrepancy, the paper version of questionnaire was cross-checked and necessary corrections made for both facility and KAP survey.

Continuous variables were tested for the assumption of normality and were presented as mean and standard deviation or median and interquartile range (IQR) where appropriate. Categorical variables were presented as frequencies and percentages. For knowledge, a correct response was scored = 1, an incorrect response = 0. In this study, we describe formal training as training on care of older persons and define recent as within the last two years [29]. Attitudes measured using a 4-point Likert scale were scored as strongly agree = 1, agree = 0.75, disagree = 0.5, strongly disagree = 0.25. Questions on practice were scored from never to sometimes to always were scored as always = 1, sometimes = 0.5, never = 0. Median score and interquartile range were calculated. In assessing practice, decision making capacity refers to the ability of the individual to give informed consent and to arrive at a course of action after review of available information [30]. A comparison between the respondent’s knowledge attitude and practice scores, and their personal characteristics were analyzed. Due to non-normality of the data, Mann-Whitney and Kruskal-Wallis tests were both used to detect significant variability in the selected variables. All tests were carried out at 5% level of significance.

Statistical analysis was conducted using STATA version 15 (StataCorp, College Station, Texas, USA).

### Ethical considerations

Ethical approval for this study was obtained from the Health Research and Ethics Committee (HREC) of the College of Medicine, University of Lagos (CMUL/HREC/10/21/954). Written informed consent was obtained from each participant and confidentiality was ensured with the use of identification codes only. The participants were informed of their right to withdraw from the study at any point in time without any consequences. No compensation was given to the participants.

## Results

### Characteristics of participants

A total of 120 health providers from the 15 comprehensive PHCs participated in this study. The mean age of the workers was 39 years, the majority female (82.5%) and about a third (34.2%) nurses (Table 1). Almost all (97.5%) of the workers had a post-secondary education and 32.7% had worked in their current role for between 10 and 20 years. Most of the workers (81.7%) had experience caring for older people but only 10.8% had a formal training in care for aging adults in the last two years (**Table 1).**

**Table 1:** Characteristics of the study participants.

Variables.	n = 120 (%)
**Age group**	
20–39	72 (60.0)
40–59	48 (40.0)
Mean age ± SD	38.6±9.3
**Sex**	
Male	21 (17.5)
Female	99 (82.5)
**Level of Education**	
Secondary	3 (2.5)
Post-secondary	117(97.5)
**Job Role**	
Medical officer	21 (17.5)
Nurse	41 (34.2)
Community health officer	15 (12.5)
Community health extension worker	35 (29.1)
*Others	8 (6.7)
**Duration in current role (Years)**	
< 10	73 (60.8)
10–20	33 (27.5)
> 20	14 (11.7)
**Any experience providing care for older people (≥60 years)**	
Yes	98 (81.7)
No	22 (18.3)
**Average No. of older persons provided care for per week (n = 98)**	
< 10	46 (46.9)
10–20	32 (32.7)
> 20	20 (20.4)
**Personal experience caring for an older family member**	113(94.2)
**Formal training on the care of older persons in the last 2 years**	13 (10.8)

*Laboratory Scientists and Pharmacists

### Knowledge of aging and AFHS

The majority (81.7%) of participants had heard about healthy aging, and most providers were aware that older people were concerned about relationships with family/friends (95.0%) and that physical strength declined in old age (90.0%) (Table 2). Similarly, most knew about factors affecting healthy aging, including the environment (90.0%), physical diseases (89.2%), lifestyle (89.2%) and social support (78.3%). Less was known about specific health conditions which affect people as they age, including depression (37.5%), urinary incontinence (35.0%), and falls/immobility (33.3%). Only 5.0% (6) had heard about age-friendly services for older adults.

**Table 2:** Knowledge of aging and age-friendly health services among participants.

Variables	n = 120 (%)
Ever heard about healthy aging (“yes”)	98 (81.7)
Aging is a disease (correctly identified “no”)	102 (85.0)
All older people become senile (correctly identified “no”)	94 (78.3)
Most older people (>60 years) can carry out their own activities (correctly identified “yes”)	93 (77.5)
All older people will become weak, frail, ill or disabled (correctly identified “no”)	106 (88.3)
Older people are all alike (correctly identified “no”)	107 (89.2)
Older people are concerned about relationships with family and friends (correctly identified “yes”)	114 (95.0)
Physical strength declines in old age (correctly identified “yes”)	108 (90.0)
Older people are unproductive and a burden to their family and society (correctly identified “no”)	86 (71.7)
**Factors affecting healthy aging** (correctly identified)	
Heredity	102 (85.0)
Activity/lifestyle	107 (89.2)
Food	102(85.0)
Physical diseases	107(89.2)
Environment	108(90.0)
Social Support	94 (78.3)
**Four main categories of impairment/chronic diseases in older people** (correctly identified)	
Fall/Immobility	40 (33.3)
Memory loss	90 (75.0)
Urinary incontinence	45 (37.5)
Depression	42 (35.0)
**Ever heard about age-friendly services for older adults (“yes”)**	6 (5.0)

### Attitude and practice of AFHS

The health providers agreed or strongly agreed on the need for accommodating and providing appropriate care for older people, with highest percent (63.3%) stating the need for compassionate staff in caring for older persons and that such care requires specific training in skills and knowledge (59.2%). About a third (32.5%) strongly agreed that the facility should provide home health services to older persons who needed it and that the models of care of the facility should support the specific needs of older people. AFHS with the lowest reported presence was access to information on evidence-based practice for care of their older patients (25.8%) and being trained in care oriented to the needs of older adults (23.3%). Over half of the providers (54.2%) screened for malnutrition in older patients, about a quarter (25.8%) screened for suspected elder abuse and much less (19.2%) for delirium in **Table 3**.

**Table 3:** Attitude and practice of AFHS among participants (n = 120).

Attitude towards AFHS	Strongly Agree	Agree	Disagree/ (Strongly)
	n (%)	n (%)	n (%)
Health facility should give priority to providing services to older persons at all points of services	65 (54.2)	51 (42.5)	4 (3.3)
If needed by an individual, the facility should provide home health services to older persons.	39 (32.5)	70(58.3)	11 (9.2)
It is important that the models of care of the facility support the specific needs of older people.	39 (32.5)	81(67.5)	0 (0.0)
Providing care to older persons requires specific training in skills and knowledge for staff	71 (59.2)	46(38.3)	3 (2.5)
Providing care to older persons requires compassionate staff	76 (63.3)	43(35.8)	1 (0.8)
Primary care facilities should have strategic commitment to care of the older person	52 (43.3)	66(55)	2 (1.7)
Primary Care facilities should have clinical providers who advocate for older people within the facility	50 (41.7)	69(57.5)	1 (0.8)
There should be robust links with relevant community organizations to facilitate care of older persons between the facility and community	58 (48.3)	61(50.8)	1 (0.8)
**Practice of AFHS**	**Always**	**Sometimes**	**Never**
Support older people in their involvement in decision making and care planning of themselves	61 (50.8)	55 (45.8)	4 (3.3)
Identify impaired capacity in decision making in older individuals	35(29.2)	80 (66.7)	5 (4.2)
Include people the older person wants involved in their care and decision making	72 (60.0)	44 (36.7)	4 (3.3)
Identify and follow older people’s advance care plans	46 (38.3)	61 (50.8)	13 (10.8)
Provide opportunities for older people to provide feedback about their care	79 (65.9)	37 (30.8)	4 (3.3)
Trained regarding older person’s sensitivities	28 (23.3)	90 (75.0)	2 (1.7)
Access to information that is clinical evidence on the care of older patients	31 (25.8)	48 (40.0)	41 (34.2)
Provide integrated assessment of the older person during the visit	45 (37.5)	51 (42.5)	24 (20.0)
Assess/consideration of non-hospital options for older patients	43 (35.8)	47 (39.2)	30 (25.0)
**Have a process to identify conditions when providing care to older adults**			
Delirium	23 (19.2)	68 (56.7)	29 (24.1)
Functional decline	35 (29.1)	80 (66.7)	5 (4.2)
Malnutrition	65 (54.2)	47 (39.1)	8 (6.7)
Incontinence	49 (40.8)	52 (43.4)	19 (15.8)
Adverse drug events	43 (35.8)	59 (49.2)	18 (15.0)
Polypharmacy	40 (33.3)	51 (42.5)	29 (24.2)
Depression	44 (36.7)	59 (49.2)	17 (14.1)
Falls	42 (35.0)	63 (52.5)	15 (12.5)
Pressure injuries	44 (36.7)	52 (43.3)	24 (20.0)
Suspected elder abuse	31 (25.8)	55 (45.8)	34 (28.4)
**Coordination of referrals for older people requiring additional care**	72 (60.0)	37 (30.8)	11 (9.2)

### Association between participants characteristics and knowledge, attitude and practice (KAP) of AFHS

The participants had a median knowledge score of 9 (IQR = 10.0) out of a total obtainable score of 44, a median attitude score of 28 (IQR = 3.0) out of 32 and a median practice score of 22 (IQR = 13.8) out of 56. The participants who had a higher level of education significantly scored better in AFHS practice (p<0.05). Those who were medical officers, had a higher level of education, who had experience caring for older people, or had a formal training in the care of older persons scored higher in their knowledge of AFHS, but it did not achieve statistical significance (p>0.05) in Table 4 and S1 Table.

**Table 4:** Association between participants characteristics and KAP score of AFHS (n = 120).

Variable	Knowledge Score (44)	Attitude Score (32)	Practice Score (56)
	Median (IQR)	Median (IQR)	Median (IQR)
**Age group**			
20–39	9.5 (7.0)	28 (3.0)	26 (14.0)
40–59	7.5 (12.0)	28 (3.0)	29 (14.8)
**p-value**	0.516	0.662	0.155
**Sex**			
Male	10 (10.0)	28 (4.0)	25 (17.5)
Female	9 (9.0)	28 (3.0)	26 (13.0)
**p-value**	0.683	0.735	0.735
**Level of Education**			
Secondary	15 (8.0)	29 (0)	17 (0)
Post-secondary	8 (9.5)	27(3.0)	27 (13.5)
**p-value**	0.052	0.069	0.033*
**Job Role**			
Medical officer	12 (8.5)	29 (3.3)	22 (14.5)
Nurse	10 (11.0)	28 (3.5)	27 (15.8)
Community health officer	7 (8.5)	27 (3.0)	26 (13.0)
Community health extension worker	7 (11.3)	28 (3.8)	30 (13.5)
Others	8 (10.0)	27 (4.5)	28 (15.0)
**p-value**	0.338	0.124	0.169
**Duration in current role (Years)**			
< 10	10 (8.3)	28 (4.0)	22 (7.0)
10–20	7 (11.3)	28 (3.0)	22 (6.5)
> 20	0 (0.0)	28 (0.0)	0 (0.0)
**p-value**	0.325	0.958	0.040*
**Any experience providing care for older people (≥60 years)**			
Yes	10 (9.3)	28 (4.0)	26 (13.3)
No	7 (12.3)	28 (2.3)	30 (19.0)
**p-value**	0.107	0.446	0.030*
**Average No. of older persons provided care for per week (n = 98)**			
< 10	9 (9.3)	27 (4.3)	21 (5.0)
10–20	10 (9.5)	28 (4.3)	22 (6.3)
> 20	10 (9.5)	27 (3.5)	25 (6.0)
**p-value**	0.946	0.643	0.277
**Personal experience caring for an older family member**			
Yes	9 (9.5)	28 (3.0)	26 (13.5)
No	12 (11.0)	28 (5.0)	29 (20.0)
**p-value**	0.686	0.561	0.658
**Formal training on the care of older persons in the last 2 years**			
Yes	12 (11.5)	28 (4.0)	26 (15.0)
No	8 (9)	28 (3.0)	26 (14.0)
**p-value**	0.160	0.625	0.065
**Overall Median Score**	**9 (IQR = 10.0)**	**28 (IQR = 3.0)**	**26 (IQR = 13.8)**

### Facility readiness for the delivery of AFHS

Medical officers in eight of 15 PHCs (46.7%) had not received any training for the care of older persons in the last two years while only two facilities (13.3%) had community health extension workers who had all been trained. Two-thirds (66.7%) of the PHCs had health workers to help older adults at the clinic however none had specially designated sections for consultations for older adults. Two-thirds (66.7%) had a functional ambulance that could be used for transport of older persons while (20.0%) of the PHCs provided home health services for older persons if required. Blood pressure measurement was always available in all the PHCs (100.0%) while only 13.3% of PHCs sometimes offered hearing assessment and none of the PHCs offered colorectal cancer assessment. One in five (20.0%) PHCs had outpatient registers with age-disaggregation for older adults and only 0.7% (n = 1) had budgets for providing care specifically for older persons **(Table 5 and S2 Table)**.

**Table 5:** Facility readiness for AFHS for older persons in PHCs (n = 15).

Variables	n (%)	n (%)	n (%)
**Health workforce training for care of older persons (in the last 2 years)**	**Yes, all**	**Yes, some**	**No**
Medical officers of Health	1 (6.7)	6 (40.0)	8 (53.3)
Medical Officers	1 (6.7)	7 (46.7)	7 (46.7)
Nurses	1 (6.7)	12 (80.0)	2 ((13.3)
Midwives	1 (6.7)	11 (73.3)	3 (20.0)
Community health officer	1 (6.7)	10 (66.7)	4 (26.7)
Community health extension worker	2(13.3)	10 (66.7)	3 (20.0)
Pharmacists	1 (6.7)	10 (66.7)	4 (26.7)
**Health service delivery**	**Yes, all**	**Yes, some**	**No**
Appointment reminders to all patients using the facility	6 (40.0)	4 (26.7)	5 (33.3)
System for giving appointment reminders to older adults using the facility	7 (46.7)	3 (20.0)	5 (33.3)
	**Yes**	**No**	
The facility has health workers to help older adults at the clinic	10 (66.7)	5 (33.3)	
Older adults have specially designated sections for consultations within the health facility	0 (0.0)	15 (100.0)	
Priority services are provided to older adults at the laboratory	12 (80.0)	3 (20.0)	
System to prioritize older adults to collect drugs	13 (86.7)	2 (13.3)	
**Availability of medical products, clinical services and essential medicines**	**Yes**	**No**	
Facility equipped with an emergency resuscitation kit for older adults	13 (86.7)	2 (13.3)	
The facility has a functional ambulance that can be used for transport of older persons	10 (66.7)	5 (33.3)	
The facility provides home health services for older persons if required	3 (20.0)	12 (80.0)	
**Availability of screening for conditions common in older adults**	**Always**	**Sometimes**	**Not at all**
Blood pressure measurement	15 (100)	0 (0.0)	0 (0.0)
Body mass index measurement	7 (46.7)	2 (13.3)	6 (40.0)
Cholesterol testing	4 (26.7)	6 (40.0)	5 (33.3)
Blood sugar check	11(78.6)	2 (13.3)	2 (13.3)
Lipid profile check	4 (26.7)	11 (73.3)	0 (0.0)
Vision screening by visual acuity	5 (33.3)	4 (26.7)	6 (40.0)
Hearing assessment	0 (0.0)	2 (13.3)	13 (86.7)
Urinary incontinence assessment	2 (13.3)	5 (33.3)	8 (53.4)
Prostate cancer assessment	1 (6.7)	3 (20.0)	11 (73.3)
Cervical cancer screening	10 (66.7)	2 (13.5)	3 (20.0)
Colorectal cancer assessment	0 (0.0)	0 (0.0)	15 (100.0)
Clinical breast examination	6 (46.2)	6 (46.2)	1 (7.7)
**Health information system**	**Yes**	**No**	
Outpatient registers with age-disaggregation that highlights for categories ≥60 years	3 (20.0)	12 (80.0)	
Laboratory registers with age-disaggregation that highlights for categories ≥60 years	15 (100.0)	0 (0.0)	
Health facility reports older persons data through DHIS 2	3 (20.0)	12 (80.0)	
Care of the older persons-focused project data by the health facility	1 (6.7)	14 (93.3)	
**Health financing for older adults**	**Yes**	**No**	
Facility budget for providing care specifically for older persons	1 (0.7)	14 (99.3)	
System for providing reductions in the cost of care on the cost of services to older persons	4 (26.7)	11 (73.3)	

### Facility design: Accessibility, physical environment and signages

Ramps were at the entrance in 60.0% of assessed PHCs but only 22.2% of those had gentle slopes which are criteria for AFHS. Almost half (43.3%) of the PHCs had the entrance to the public toilets accessible to wheelchair users but only 13.3% of the toilets had grab bars around them. Identification of individuals working in the PHCs was low, 20.0% had a posted board that included all staff with the job title on duty and name tags were used by staff in only one centre **(Table 6)**.

**Table 6:** Facility design: Accessibility, physical environment and signages (n = 15).

Variables	n (%)	n (%)
Accessibility	Yes	No
Center served by public transportation	10 (66.7)	5 (33.3)
Signs at main lobbies or main traffic routes to PHC	9 (60.0)	6 (40.0)
Ramp at the entrance	9 (60.0)	6 (40.0)
Ramp have a slope greater than (1:12)	2(22.2)	7 (77.8)
Width of the entrance greater than or equal to 900 mm	6 (40.0)	9 (60.0)
Parking lot with spaces dedicated for disabled or older person near the main entrance	0 (0.0)	15 (100)
Reception near the entrance	10 (66.7)	5 (33.3)
All doors width greater than or equal to 900 mm	8 (53.3)	7 (46.7)
**Safety of physical environment**		
In patient areas, with floor non-slippery	15 (100)	0 (0.0)
Furniture and fittings in the reception well organized to reduce possible falls or injuries	14 (93.3)	1 (6.7)
Toilets near the out-patient waiting area	11 (73.3)	4 (26.6)
Entrance to the public toilet accessible to wheelchair users	8 (53.3)	7 (46.7)
Grab bars around the toilet	2 (13.3)	13 (86.7)
Corridor have minimum unobstructed width for wheelchair users	15 (100)	0 (0.0)
**Signages**		
Characters and symbols contrast with their background	10 (66.7)	5 (33.3)
Visual displays simple and easy to understand	11 (73.3)	4 (26.7)
Common and familiar pictures to the community used whenever possible	6 (40.0)	9 (60.0)
Tone of the sign messages is welcoming and cordial	11 (73.3)	4 (26.7)
Signs placed at eye level	12 (80.0)	3 (20.0)
Signs outside the building to identify buildings within the facility compound	10 (66.7)	5 (33.3)
**Identification of personnel**		
PHC centre staff are easily identifiable using name tags	1 (6.7)	14 (93.3)
Name board that includes all staff with job title on duty	3 (20.0)	12 (80.0)

## Discussion

We found that while there was considerable interest and appreciation of the need to deliver care designed for older people, there were significant gaps in readiness at the facility and individual HCW levels. The study found inadequate training on the care of older persons among the primary health care workers but a high level of interest and a positive attitude towards the need for aging services in PHCs. There were significant challenges in infrastructure, service delivery and care oriented to the needs of older adults. By using the WHO’s guideline for age-friendly PHCs to assess facility and provider readiness, we have been able to demonstrate its usefulness in identifying gaps in the PHCs and to determine specific areas of focus for health systems in the provision of age-friendly services. The study found that the given the gaps in readiness in the infrastructure HCW knowledge and experience and availability of services, that the current PHC in Lagos Nigeria may not be positioned to provide the quality care to older adults needed to achieve the targeted expected benefits of healthy aging in its population [15, 31]. Some of the challenges highlighted for action in the strategic roadmap framework (2022–2032) for the implementation of the National Policy on Ageing 2021 include; the lack of geriatric services, trained geriatricians and age-friendly health facilities. In addition, the absence of awareness and training on age-friendliness at the PHC centers, lack of healthcare insurance for older people and health facilities that do not accommodate the accessibility needs of older adults [11].

We found that PHC staff had been in place for significant time, and provided care to older individuals, and there was good awareness on aging and factors affecting aging but low on the knowledge of the key conditions which threaten healthy aging including falls, depression and urinary incontinence. This low level of age-friendly knowledge is consistent with other studies of care providers in clinic settings in Benin City, Nigeria [21] Uganda [20] and the United States [19, 32]. There were no comparable studies in similar settings to ours within our search.

The attitude of health care providers towards older adults and the provision of age-friendly services is of growing importance globally with the aging of populations in many countries [33]. The health providers in this study had a positive attitude towards the need for accommodating and providing appropriate care for older people. This is consistent with the findings from a systematic review of health professionals whose attitude towards older patients ranged from neutral to positive in China [33].

Given Nigeria’s rapidly aging population, it is expected that the demand for home-based care will increase significantly and care support will be required. However, we found that only about a third of health workers strongly agreed to the facility providing home health services to older persons. This reluctance may be as a result of the reduced capacity and shortage of health care workers across PHCs in Nigeria attributed to lack of regular recruitment processes [34]. A similar proportion of participants strongly agreed on the importance of models of care that support the specific needs of older people. It is documented that older adults with multi-morbidities and chronic conditions have unmet care needs in their physical, mental, social health and the environment that require the development of care models and support services based on their needs [13].

The practice of some screening important for aging populations was also found to be low. These included identification of impaired decision-making capacity in older individuals. The study among family members and dependent older people in Nigeria, China, Mexico and Peru found there were decision making needs for the care of their dependent older adults and often left it to the males in the family [35]. This decision-making capacity in older adults is increasingly important due to aging populations and dementia-related diseases [36]. Training of health providers in care oriented to older people also scored low in this study. Less than 13.3% of the facilities surveyed had all the health workers in some cadre trained in the last two years. This does not seem unusual as primary health care professionals in a study in Greece found that participants had not received any formal training in geriatric medicine [37].

According to our findings, health providers who were better educated were more likely to have better AFHS practice (p<0.05). Similarly, medical officers scored highest in AFHS knowledge and attitude among the health providers followed by nurses and community health officers. Geriatric medicine is an emerging subspecialty in Nigeria with the establishment of the pioneer geriatric centre in Nigeria in 2012 [38]. For example, the University College Hospital, Ibadan, Nigeria, offers a 2-week basic certificate course in geriatric medicine but this training is for doctors [39]. This may account for the deficiency in training among the primary care providers. Training on care of older persons is critical at multiple levels including the patient, the provider and the health system to address the issues of health care access, quality of care and multicomplexity [40].

In age-friendly health service delivery, about two-thirds of the facilities had health workers to help older adults at the clinic and 4 in 5 provided priority services to older adults at the laboratory but none had specially designated sections for consultations for older adults within the health facility. Pharmacies in Iranian PHCs were found to give priority to seniors in collecting drugs, but this service was spontaneous provided without any special protocol and guidelines [41]. In some cases, the assistance rendered to older adults in the African setting conforms with the norms of respect for older people. Older adults often have special needs and chronic conditions that require care improved primary care with stronger processes of coordination and enhanced patient engagement [42, 43].

While the health management information system captured age, we found that only 3 of the facilities had age-disaggregation for older adults 60 years and above in the outpatient registers and only one facility had care of the older persons-focused project data. It has been noted that countries use different age groupings when reporting health data and this lack of standardized age disaggregation became evident during the COVID-19 pandemic [44]. WHO recommends that groupings for the age standardization use 5-year groups from birth up to the oldest reached age (100 years or older) [45]. Among the Ten Plus One (10 +1) policy goals for the National Policy on Ageing 2021, goal 11 aims to facilitate the production of comprehensive and disaggregated data [11].

Health financing for older adults was inadequate with only one facility in our study budgeting for providing care for older persons. Although the national health policies strongly recommend Local Governmental Authorities (LGAs) establish a budget-line for PHC, the operationalization to finance key health priorities in the PHC greatly depends on LGA chairmen and their political will [46]. Older adults are not included in the National Health Insurance Scheme (NHIS) and out-of-pocket payments (OOP) are the most prevalent method of financing health care costs with as much as 96% of private health expenditure made through OOP in Nigeria [47, 48].

Physical access is an important component of age-friendly health care centres in consideration of older adults with mobility, hearing or visual impairments [14]. Two-thirds of the facilities visited were served by public transport and 60% had signs at the entrance or main routes to the PHCs but none had parking spaces dedicated for disabled or older persons. This finding is consistent with the study in Iran [41]. Although 9 out of the 15 facilities surveyed had ramps for wheelchair users at the entrance, less than 14 out of 15 PHCs that were wheelchair accessible in Saudi Arabia [49], however, only 2 of the ramps in our surveyed PHCs had the gentle slope ratio as recommended by WHO [14]. There was a safe physical environment in most of the facilities visited as all had non-slippery floors and unobstructed corridors for use by wheelchair users however, access to the toilets by wheelchair users was only available in half of the facilities and only 13.3% of them had grab bars around the toilet. This finding is lower than in 40.0% of the facilities with grab bars in Saudi Arabia [49]. Falls causing injury in older adults have been documented to occur most commonly in the bathroom due to deteriorating levels of mobility and sense of balance. Such risk is higher in the absence of grab bars for the toilets and bathtubs [50, 51]. Availability and good practice of signage designs ranged from 40–80% in the facilities visited similar to the Iran study [51]. Signages is important in wayfinding, providing reassurance and helping to maintain high mobility for older adults [52].

There are some limitations to be considered while interpreting the findings from this study. First, the responses from the participants to ‘agree’ or ‘strongly agree’ to the various attitude statements may have been impacted by cultural factors due to the value placed on older persons in the Nigerian culture. Second, our study sample may not be representative of health care workers in PHCs in Lagos, Nigeria, next, there is the possibility for social desirability bias from the responses and we attempted to limit this by conducting the interviews in private areas. Understanding of barriers and facilitators was also not included although work is ongoing to analyze qualitative interviews to explore these factors. Finally, we did not include aging population in this study, so experiences of care are not captured.

In conclusion, this study across cadres of providers in Lagos State PHCs were found to be knowledgeable about healthy aging and the need for healthy aging services but low on awareness on age-friendly services and training gaps were identified. This study also identified significant challenges in some aspects of screening for conditions common in older adults, infrastructure and facility design in most of the PHCs. These results identified opportunities for strengthening capacity and future research should focus on the needs, expectations and challenges of older adults in accessing the PHCs in Lagos, Nigeria and interventions to improve the availability and delivery of care.

## Supporting information

S1 Table Association between participants’ characteristics and knowledge, attitude and practice of age-friendly health services. (DOCX)

S2 Table Facility readiness for AFHS for older persons in PHCs (n = 15). (DOCX)

S1 Data Study dataset. (XLSX)
