# Supplementary material for: Provider and facility readiness for age-friendly health services for older adults in primary health care centres in southwest, Nigeria
Source: PLOS Glob Public Health. 2023 Aug 8;3(8):e0001411. doi: 10.1371/journal.pgph.0001411 (PMC10409274; doi:10.1371/journal.pgph.0001411)
Supplement: S1 Table — (DOCX) [file pgph.0001411.s001.docx]

**S1 Table-Association between participants’ characteristics and knowledge, attitude and practice of age-friendly health services**

|  | **Knowledge** | | **Attitude** | | **Practice** | |
| --- | --- | --- | --- | --- | --- | --- |
| **Variable** | **Coefficient** | **Sig.** | **Coefficient** | **Sig.** | **Coefficient** | **Sig.** |
| Age | 0.02 | 0.87 | -0.04 | 0.33 | 0.16 | 0.25 |
| Gender | 0.17 | 0.11 | 0.03 | 0.76 | 0.03 | 0.75 |
| Education | -0.15 | 0.13 | 0.19 | 0.08 | -0.18 | 0.09 |
| Job Role | -0.19 | 0.07 | 0.18 | 0.10 | -0.20 | 0.07 |
| Years worked in the current role (years). | -0.25 | 0.08 | 0.14 | 0.33 | -0.14 | 0.34 |
| Experienced caring for people ≥60 years | 0.12 | 0.19 | -0.62 | 0.54 | -0.26 | 0.00* |
| Average no. of older persons provided care for per week | 0.03 | 0.76 | -0.07 | 0.52 | 0.17 | 0.12 |
| Personal experience caring for an older family member ≥60 years | -0.21 | 0.04* | 0.10 | 0.37 | -0.08 | 0.43 |
| Formal training related to the care for older persons in the last 2 years. | 0.09 | 0.41 | 0.6 | 0.56 | -0.19 | 0.07 |
| *significant at p–value <0.05 | | | | | | |
