# Supplementary material for: Provider and facility readiness for age-friendly health services for older adults in primary health care centres in southwest, Nigeria
Source: PLOS Glob Public Health. 2023 Aug 8;3(8):e0001411. doi: 10.1371/journal.pgph.0001411 (PMC10409274; doi:10.1371/journal.pgph.0001411)
Supplement: S2 Table — (DOCX) [file pgph.0001411.s002.docx]

**S2 Table-Facility readiness for AFHS for older persons in PHCs (n=15)**

| **Variables** | **n (%)** | **n (%)** | **n (%)** | **n (%)** |
| --- | --- | --- | --- | --- |
| **Availability of health and lifestyle counselling services** |  | **Always** | **Sometimes** | **Not at all** |
| Calcium intake |  | 9 (60.0) | 6 (40.0) | 0 (0.0) |
| Tobacco cessation |  | 8 (53.3) | 7 (46.7) | 0 (0.0) |
| Drug and alcohol use |  | 7 (46.7) | 8 (53.3) | 0 (0.0) |
| Healthy eating |  | 11 (73.3) | 4 (26.7) | 0 (0.0) |
| Physical activity |  | 11 (73.3) | 4 (26.7) | 0 (0.0) |
| Oral health |  | 9 (60.0) | 6 (40.0) | 0 (0.0) |
| Injury/fall prevention |  | 10 (66.7) | 5 (33.3) | 0 (0.0) |
| Polypharmacy |  | 9 (60.0) | 5 (33.3) | 1 (6.7) |
| **Availability of essential medicines for older adults** |  | **Always** | **Sometimes** | **Not at all** |
| Nutritional supplements- Calcium, Vit. D, Zinc |  | 14 (93.3) | 0 (0.0) | 1 (6.7) |
| Anti-depressants |  | 9 (60.0) | 0 (0.0) | 6 (40.0) |
| Treatment for urinary incontinence |  | 5 (33.3) | 2 (13.3) | 8 (53.3) |
| Treatment for insomnia |  | 13 (100) | 2 (13.3) | 0 (0.0) |
| **Availability of tests, equipment and commodities** | **Seen/**  **functional** | **Seen/not functional** | **Reported, not seen** | **Not available** |
| Microscope | 15 (100) |  |  |  |
| Glucometer | 14 (93.3) | 1 (6.7) | 0 (0.0) | 0 (0.0) |
| Urinalysis strips | 15 (100) | 0 (0.0) | 0 (0.0) | 0 (0.0) |
| Thermometer | 15 (100) | 0 (0.0) | 0 (0.0) | 0 (0.0) |
| Wheel chair | 9 (60.0) | 0 (0.0) | 3 (20.0) | 3 (20.0) |
| Oxygen cylinder | 6 (40.0) | 2 (13.3) | 6 (40.0) | 1 (6.7) |
| Visual Acuity screening chart | 8 (53.3) | 0 (0.0) | 1 (6.7) | 6 (40.0) |
